# Chronic kidney disease and its association with cerebral small vessel disease in the general older hypertensive population

**DOI:** 10.1186/s12882-024-03528-8

**Published:** 2024-03-13

**Authors:** Tomas Månsson, Aldana Rosso, Katarina Ellström, Kasim Abul-Kasim, Sölve Elmståhl

**Affiliations:** 1 Department of Clinical Sciences in Malmö, Division of Geriatric Medicine, Lund University and Skåne University Hospital, Jan Waldenströms gata 35, pl 13, 205 02 Malmö, Sweden; 2 Department of Clinical Sciences in Lund, Division of Diagnostic Radiology, Lund University, 221 85 Lund, Sweden

**Keywords:** Kidney function, Glomerular filtration rate, Cerebral small vessel disease, Hypertension, White matter hyperintensities, Lacunar infarcts, Cerebral microbleeds, Cortical atrophy

## Abstract

**Background:**

Cerebral small vessel disease can be identified using magnetic resonance imaging, and includes white matter hyperintensities, lacunar infarcts, cerebral microbleeds, and brain atrophy. Cerebral small vessel disease and chronic kidney disease share many risk factors, including hypertension. This study aims to explore an association between chronic kidney disease and cerebral small vessel disease, and also to explore the role of hypertension in this relationship.

**Methods:**

With a cross sectional study design, data from 390 older adults was retrieved from the general population study Good Aging in Skåne. Chronic kidney disease was defined as glomerular filtration rate < 60 ml/min/1,73m^2^. Associations between chronic kidney disease and magnetic resonance imaging markers of cerebral small vessel disease were explored using logistic regression models adjusted for age and sex. In a secondary analysis, the same calculations were performed with the study sample stratified based on hypertension status.

**Results:**

In the whole group, adjusted for age and sex, chronic kidney disease was not associated with any markers of cerebral small vessel disease. After stratification by hypertension status and adjusted for age and sex, we observed that chronic kidney disease was associated with cerebral microbleeds (OR 1.93, CI 1.04–3.59, p-value 0.037), as well as with cortical atrophy (OR 2.45, CI 1.34–4.48, p-value 0.004) only in the hypertensive group. In the non-hypertensive group, no associations were observed.

**Conclusions:**

In this exploratory cross-sectional study, we observed that chronic kidney disease was associated with markers of cerebral small vessel disease only in the hypertensive subgroup of a general population of older adults. This might indicate that hypertension is an important link between chronic kidney disease and cerebral small vessel disease. Further studies investigating the relationship between CKD, CSVD, and hypertension are warranted.

## Background

Cerebral small vessel disease (CSVD) includes pathologies of the small vessels in the brain, including small arteries, arterioles, venules, and capillaries. These pathologies are diagnosed and identified on magnetic resonance imaging (MRI) as white matter hyperintensities (WMHs), lacunar infarcts, cerebral microbleeds (CMBs), brain atrophy, recent small subcortical infarcts, and perivascular spaces. CSVD is an important cause of cognitive decline, dementia, stroke, and functional impairment [1, 2]. Although the prevalence of CSVD varies between studies, it increases with age and is very common in the older population [3, 4]. For example, the prevalence of CMBs has been estimated to increase from 7% in people 45–50 years to 36% in people ≥ 80 years [5]. The prevalence of lacunar infarcts has been estimated to increase from 22% at age 75 years to 32% at age 80 years [6].

Chronic kidney disease (CKD) is related to age, and it is– like CSVD– also prevalent in the older population [7]. Previously, we and others have shown an association between CKD and cognitive impairment [8, 9]. In our previous work, we found a longitudinal association between CKD and the development of impairment in processing speed [10]. Processing speed and executive function seem to be more affected by CSVD, compared to other cognitive domains [11, 12]. The kidney and the brain share similar low vascular resistance mechanisms, presumably making both organs sensible to hypertension [13, 14]. CKD is related to other vascular diseases, such as myocardial infarction and intermittent claudication [15, 16]. These findings suggest that an association between CKD and CSVD is possible.

Studies investigating the relationship between CKD and CSVD have found mixed results. In a pooled meta-analysis published in 2023 (*n* = 57 030), Xiao et al. [17] found CKD (eGFR < 60 ml/min/1,73m^2^) to be associated with WMHs (CI 1.05–1.86), lacunar infarcts (CI 1.18–1.92), and CMBs (CI 1.26–1.90), but not with perivascular spaces (CI 0.77–1.88). Similar results were noted in another pooled meta-analysis (*n* = 10 534) [18], where low eGFR was associated with WMHs, lacunar infarcts, CMBs, and silent cerebral infarction. Interestingly, in another meta-analysis (*n* = 20 379), Makin et al. [19] found lacunar infarcts not to be associated with CKD (defined as eGFR < 60 ml/min/1,73m^2^ or presence of albuminuria). In summary, CKD was associated with WMHs and CMBs in two large meta-analyses. One large meta-analysis found an association between CKD and lacunar infarcts, while another did not. There are relatively few studies that have investigated the relationship between CKD and brain atrophy. Vogels et al. [20] identified 3 studies investigating an association between eGFR and brain atrophy, in which all studies found such an association. Since previous studies investigating a relationship between CKD and markers of CSVD have found conflicting results, and studies investigating the relationship between CKD and brain atrophy are lacking, further studies investigating the relationship between CKD and CSVD are indicated.

CKD and CSVD share many of the same risk factors, including hypertension [13]. The prevalence of hypertension has been estimated to be as high as 65% in individuals ≥ 60 years of age [21]. This probably makes hypertension the most common treatable risk factor of both CKD and CSVD, and therefore the most important. The aims of this study were to explore an association between CKD and CSVD, as well as the role of hypertension in this possible association.

## Methods

### Study population

All data used in this cross-sectionally designed study was collected from the ongoing population-based cohort study “Good Aging in Skåne” (GÅS), conducted at the Department of Geriatric Medicine, Skåne University Hospital in Malmö, Sweden [22]. GÅS is part of the “Swedish National study on Aging and Care” (SNAC) [23]. In GÅS, participants ≥ 60 years of age have randomly been recruited from the population register since 2001. Participants represent the general, older population from the south part of Sweden. Participants in the GÅS study are invited to be examined every 3–6 years, depending on age, until death. The survey includes physical and medical examination, medical history, interview, anthropometrics, neuropsychological testing, blood sampling, and self-reported questionnaires. New participants have been recruited continuously to the GÅS study, following the same procedure as described above. A more thorough description of GÅS has been presented elsewhere [22].

A subgroup of 407 participants underwent MRI examination between March 2016 to March 2018. Out of these, 17 had missing blood samples, leaving 390 participants in this study.

### Kidney function

Participants are invited for re-examinations in a nonfasted state every 3–6 years. Cryopreserved blood samples were collected from the nearest examination prior to the MRI examination (mean 738 (SD 366) days. Cystatin C (cysC) and creatinine (crea) were later analyzed at the Skåne University Hospital laboratory in Malmö. CysC was analyzed using Gentians reagent with a Beckman Coulter LX20 [24], crea was analyzed using a modified Jaffe method with a Beckman Coulter LX20 traceable to isotope-dilution mass spectrometry (IDMS).

CKD is often defined as estimated glomerular filtration rate (eGFR) < 60 ml/min/1.73m^2^ [25] based on the well-established and reliable chronic kidney disease epidemiology collaboration (CKD-EPI) formula, in which consideration is taken for age and sex [26]. Calculations can be made based on crea, cysC or both (crea/cysC). In this study, mean eGFR from crea/cysC was used to assess eGFR in non-underweight participants [27]. Having a body mass index (BMI) < 23 was defined as being underweight [28]. To minimize bias, the same formula was used to calculate eGFR based only on cysC in underweight participants [29]. CKD was defined as having an eGFR of < 60 ml/min/1.73m^2^. Normal kidney function was defined as having an eGFR ≥ 60 ml/min/1.73m^2^.

### Neuroradiology

MRI was performed in the Radiology Department at Skåne University Hospital in Malmö, using a 3 Tesla MRI (General Electric discovery MR 750w). The MRI examination included axial diffusion-weighted images (DWI), axial T2-weighted fluid-attenuated inversion recovery (T2 FLAIR), and axial susceptibility-weighted angiography (SWAN). An experienced neuroradiologist examined all 407 MRI examinations systematically, blinded for clinical information. The MRI examination has been described in more detail elsewhere [30].

FLAIR sequences were used to assess *WMHs*. The Fazekas scale [31] was used to grade the presence of WMHs, where a score of ≥ 2 was considered pathological and representative of pathological presence of WMHs.

*Lacunar infarcts* were defined as having ≥ 1 ischemic infarction (< 1.0 cm) in the deep white matter, the basal ganglia or pons [32].

*CMBs* were defined as having ≥ 1 small (0,2 − 0,5 cm) hypointense lesion [32], using the SWAN sequence.

*Cortical atrophy* was defined as presence of at least one of the following entities: Global cortical atrophy (GCA) ≥ 1 according to the Pasquier scale [33], Koedam score ≥ 1 [34], and/or presence of frontal/frontotemporal/temporal atrophy (visually assessed by an experienced neuroradiologist).

*The composite CSVD* variable was constituted of a modified version of the cerebral small vessel disease (SVD) score, presented by Staals et al. in 2014 [35]. Presence of CSVD according to the composite CSVD variable was defined as presence of at least one of the following: Fazekas scale ≥ 2, ≥ 1 lacunar infarct, and/or ≥ 1 CMB.

*The modified STRIVE* variable was constituted of a modified version of the Standards for reporting vascular changes on neuroimaging (STRIVE), presented by Wardlaw et al. in 2013 [36]. Presence of CSVD according to the modified STRIVE variable was defined as presence of at least one of the following: Fazekas scale ≥ 2, ≥ 1 lacunar infarct, ≥ 1 CMB, cortical atrophy, and/or central atrophy. Presence of central atrophy was visually assessed by an experienced neuroradiologist.

### Hypertension

A history of hypertension was assessed by a physician from medical history and medical record for an ICD-10 diagnosis of primary or secondary hypertension. Current use of antihypertensive treatment was documented using Anatomical Therapeutic Chemical (ATC) codes (antihypertensive agents ATC02, diuretics TC03, beta-blockers ATC07, calcium antagonists ATC08, and renin-angiotensin-system inhibitors ATC09). Medical records were also reviewed in retrospect for an ICD-10 diagnosis of primary or secondary hypertension diagnosed any time before the MRI examination.

### Statistics

Binary logistic regression models were used to explore associations between CKD (eGFR < 60 ml/min/1.73 m^2^) and the presence of WMHs, lacunar infarcts, CMBs, cortical atrophy, composite CSVD, and modified STRIVE.

First, we estimated the logistic models without covariates. Kidney function and CSVD are affected by age and sex. Therefore, to minimize confounding, we also estimated the logistic models including these covariates.

In a secondary analysis the sample was stratified by hypertension status. Binary logistic models were used to explore associations between CKD and the presence of WMHs, lacunar infarcts, CMBs, cortical atrophy, composite CSVD, and modified STRIVE. Due to limited sample size, adjustments to mitigate confounding were made including only age and sex in the statistical models. The results are presented in Table 1.

**Table 1:** Description of the study sample. Sample stratified by CKD status

	eGFR < 60 ml/min/1.73m2	eGFR ≥ 60 ml/min/1.73m2	All
Number	94	296	390
Age in years	76.6 (SD 3.6)	75.0 (SD 3.5)	75.4 (SD 3.6)
Female (%)	57 (60.6)	157 (53.0)	214 (54.9)
Years of education	10.7 (SD 3.4)	11.8 (SD 3.8)	11.5 (SD 3.7)
Smoking, current or former	60 (63.8)	176 (59.5)	236 (60.5)
BMI	27.6 (SD 4.2)	26.5 (SD 3.9)	26.7 (SD 4.0)
Diabetes, type 1 or 2 (%)	19 (20.2)	43 (14.5)	62 (15.9)
Hypertension, primary or secondary (%)	74 (78.7)	132 (44.6)	206 (52.8)
Heart disease (%)^1^	41 (43.6)	88 (29.7)	129 (33.1)
Cerebrovascular disease (%)^2^	16 (17.0)	30 (10.1)	46 (11.8)
eGFR mean (ml/min/1.73m^2^)	50.2 (SD 8.4)	77.3 (SD 10.8)	70.8 (SD 15.5)
MRI findings			
WMHs (%)^3^	37 (39.4)	83 (28.0)	120 (30.8)
Lacunar infarcts (%)^4^	10 (10.6)	22 (7.4)	32 (8.2)
CMBs (%)^5^	34 (36.2)	73 (24.7)	107 (27.4)
Cortical atrophy (%)^6^	56 (59.6)	141 (47.6)	197 (50.5)
Composite CSVD (%)^7^	58 (61.7)	140 (47.3)	198 (50.8)
Modified STRIVE (%)^8^	80 (85.1)	229 (77.4)	309 (79.2)

eGFR calculated from creatinine and cystatin C in non-underweight participants and on cystatin C in underweight participants (BMI < 23) using the CKD-EPI formula

Abbreviations: BMI = Body mass index, CKD = Chronic kidney disease, CKD-EPI = chronic kidney disease epidemiology collaboration, CMBs = Cerebral microbleeds, CSVD = Cerebral small vessel disease, eGFR = estimated glomerular filtration rate, GCA = Global cortical atrophy, MRI = Magnetic resonance imaging, SD = Standard deviation, STRIVE = Standards for reporting vascular changes on neuroimaging, WMHs = White matter hyperintensities

^1^ Coronary artery disease, heart failure, atrial fibrillation, and/or presence of cardiac and vascular implants and grafts

^2^ Nontraumatic intracranial hemorrhage, cerebral infarction, stenosis of cerebral or precerebral arteries, and/or transient ischemic attack

^3^ WMHs defined as Fazekas scale ≥ 2

^4^ Lacunar infarcts defined as presence of ≥ 1 lacunar infarct

^5^ CMBs defined as presence of ≥ 1 CMB

^6^ Cortical atrophy defined as GCA ≥ 1 and/or Koedam score ≥ 1 and/or ≥ 1 frontal/frontotemporal/temporal atrophy

^7^ Presence of CSVD according to the composite CSVD variable was defined as Fazekas scale ≥ 2 and/or presence of ≥ 1 lacunar infarct and/or ≥ 1 CMB

^8^ Presence of CSVD according to the modified STRIVE variable was defined as Fazekas scale ≥ 2 and/or presence of ≥ 1 lacunar infarct and/or presence of ≥ 1 CMB and/or presence of cortical atrophy and/or presence of central atrophy

All statistical analyses were performed using the software IBM SPSS version 27. The statistical significance level was set at 5%.

## Results

A description of the characteristics of the study sample stratified based on kidney function is presented in Table 2. In Table 3, the study sample was stratified by hypertension status.

**Table 2:** Description of the study sample. Sample stratified by hypertension status

	Hypertension	No hypertension	All
Number	206	184	390
Age in years	76.1 (SD 3.9)	74.5 (SD 3.1)	75.4 (SD 3.6)
Female (%)	110 (53.4)	104 (56.5)	214 (54.9)
Years of education	11.1 (SD 3.7)	12.0 (SD 3.6)	11.5 (SD 3.7)
Smoking, current or former (%)	126 (61.2)	110 (59.8)	236 (60.5)
BMI	27.6 (SD 4.0)	25.8 (SD 3.8)	26.7 (SD 4.0)
Diabetes, type 1 or 2 (%)	49 (23.8)	13 (7.1)	62 (15.9)
Heart disease (%)^1^	86 (41.7)	43 (23.4)	129 (33.1)
Cerebrovascular disease (%)^2^	36 (17.5)	10 (5.4)	46 (11.8)
eGFR mean (ml/min/1.73m^2^)	66.3 (SD 16.2)	75.8 (SD 13.0)	70.8 (SD 15.5)
MRI findings			
WMHs (%)^3^	80 (38.8)	40 (21.7)	120 (30.8)
Lacunar infarcts (%)^4^	23 (11.2)	9 (4.9)	32 (8.2)
CMBs (%)^5^	66 (32.0)	41 (22.3)	107 (27.4)
Cortical atrophy (%)^6^	103 (50.0)	94 (51.1)	197 (50.5)
Composite CSVD (%)^7^	122 (59.2)	76 (41.3)	198 (50.8)
Modified STRIVE (%)^8^	170 (82.5)	139 (75.5)	309 (79.2)

eGFR calculated from creatinine and cystatin C in non-underweight participants and on cystatin C in underweight participants (BMI < 23) using the CKD-EPI formula

*Abbreviation* BMI = Body mass index, CKD = Chronic kidney disease, CKD-EPI = chronic kidney disease epidemiology collaboration, CMBs = Cerebral microbleeds, CSVD = Cerebral small vessel disease, eGFR = estimated glomerular filtration rate, GCA = Global cortical atrophy, MRI = Magnetic resonance imaging, SD = Standard deviation, STRIVE = Standards for reporting vascular changes on neuroimaging, WMHs = White matter hyperintensities

^1^ Coronary artery disease, heart failure, atrial fibrillation, and/or presence of cardiac and vascular implants and grafts

^2^Nontraumatic intracranial hemorrhage, cerebral infarction, stenosis of cerebral or precerebral arteries, and/or transient ischemic attack

^3^ WMHs defined as Fazekas scale ≥ 2

^4^ Lacunar infarcts defined as presence of ≥ 1 lacunar infarct

^5^ CMBs defined as presence of ≥ 1 CMB

^6^Cortical atrophy defined as GCA ≥ 1 and/or Koedam score ≥ 1 and/or ≥ 1 frontal/frontotemporal/temporal atrophy

^7^ Presence of CSVD according to the composite CSVD variable was defined as Fazekas scale ≥ 2 and/or presence of ≥ 1 lacunar infarct and/or ≥ 1 CMB

^8^ Presence of CSVD according to the modified STRIVE variable was defined as Fazekas scale ≥ 2 and/or presence of ≥ 1 lacunar infarct and/or presence of ≥ 1 CMB and/or presence of cortical atrophy and/or presence of central atrophy

**Table 3:** Association between CKD (eGFR < 60 ml/min/1.73m^2^) and CSVD

	Odds ratio	CI	p-value
*Association between CKD and CSVD, not adjusted for covariates*			
WMHs^1^	1.67	1.03–2.71	0.039
Lacunar infarcts^2^	1.49	0.68–3.27	0.321
CMBs^3^	1.72	1.04–2.82	0.033
Cortical atrophy ^4^	1.62	1.01–2.59	0.045
Composite CSVD ^5^	1.8	1.12–2.89	0.016
Modified STRIVE ^6^	1.67	0.89–3.14	0.11
*Association between CKD and CSVD, adjusted for age and sex*			
WMHs^1^	1.44	0.88–2.37	0.152
Lacunar infarcts^2^	1.57	0.70–3.53	0.273
CMBs^3^	1.61	0.96–2.70	0.074
Cortical atrophy ^4^	1.52	0.93–2.48	0.093
Composite CSVD ^5^	1.55	0.95–2.53	0.079
Modified STRIVE ^6^	1.38	0.71–2.66	0.344

eGFR calculated from creatinine and cystatin C in non-underweight participants and on cystatin C in underweight participants (BMI < 23) using the CKD-EPI formula

eGFR ≥ 60 ml/min/1.73m^2^ as reference

Binary logistic regression models were used to examine the association between CKD (eGFR < 60 ml/min/1.73m^2^) and WMHs, lacunar infarcts, CMBs, cortical atrophy, CSVD composite, and modified STRIVE.

Covariates: Age, sex

*Abbreviation* BMI = Body mass index, CKD = Chronic kidney disease, CKD-EPI = chronic kidney disease epidemiology collaboration, CMBs = Cerebral microbleeds, CSVD = Cerebral small vessel disease, eGFR = estimated glomerular filtration rate, GCA = Global cortical atrophy, MRI = Magnetic resonance imaging, SD = Standard deviation, STRIVE = Standards for reporting vascular changes on neuroimaging, WMHs = White matter hyperintensities

^1^ WMHs defined as Fazekas scale ≥ 2

^2^ Lacunar infarcts defined as presence of ≥ 1 lacunar infarct

^3^ CMBs defined as presence of ≥ 1 CMB

^4^Cortical atrophy defined as GCA ≥ 1 and/or Koedam score ≥ 1 and/or ≥ 1 frontal/frontotemporal/temporal atrophy

^5^ Presence of CSVD according to the composite CSVD variable was defined as Fazekas scale ≥ 2 and/or presence of ≥ 1 lacunar infarct and/or ≥ 1 CMB

^6^Presence of CSVD according to the modified STRIVE variable was defined as Fazekas scale ≥ 2 and/or presence of ≥ 1 lacunar infarct and/or presence of ≥ 1 CMB and/or presence of cortical atrophy and/or presence of central atrophy

Compared to participants with normal kidney function (eGFR ≥ 60 ml/min/1.73m^2^), participants with CKD (eGFR < 60 ml/min/1.73m^2^) more often had WMHs (39.4% vs. 28.0%), lacunar infarcts (10.6% vs. 7.4%), CMBs (36.2% vs. 24.7%), and cortical atrophy (59.6% vs. 47.6%). The criteria for CSVD according to the composite CSVD variable and the modified STRIVE variable, were also more often met in the group with CKD compared to the group with normal kidney function (61.7% vs. 47.3% and 85.1% vs. 77.4%, respectively). This is presented in Table 2.

The results from both the unadjusted and adjusted models are presented in Table 4. Unadjusted, we observed that CKD was associated with WMHs, CMBs and cortical atrophy, as well as with the composite CSVD variable. However, after adjusting the statistical models for age and sex, none of the associations between CKD and CSVD remained significant.

**Table 4:** Association between CKD (eGFR < 60 ml/min/1.73m^2^) and CSVD, stratified by hypertension

Participants with hypertension (n = 206)					Participants with no hypertension (n = 184)			
*Association between CKD and CSVD, not adjusted for covariates*								
	Odds ratio	CI	p-value			Odds ratio	CI	p-value
WMHs^1^	1.22	0.68–2.18	0.501	WMHs^1^		1.64	0.59–4.58	0.346
Lacunar infarcts^2^	0.96	0.39–2.39	0.93	Lacunar infarcts^2^		2.46	0.48–12.75	0.284
CMBs^3^	1.96	1.07–3.58	0.029	CMBs^3^		0.59	0.16–2.10	0.412
Cortical atrophy ^4^	2.59	1.43–4.66	0.002	Cortical atrophy ^4^		0.61	0.24–1.56	0.297
Composite CSVD ^5^	1.58	0.88–2.86	0.127	Composite CSVD ^5^		1.19	0.47–3.02	0.722
Modified STRIVE ^6^	1.86	0.82–4.20	0.137	Modified STRIVE ^6^		0.97	0.33–2.83	0.952
*Association between CKD and CSVD, adjusted for age and sex*								
WMHs^1^	1.15	0.64–2.08	0.633	WMHs^1^		1.28	0.45–3.70	0.643
Lacunar infarcts^2^	1.11	0.44–2.82	0.825	Lacunar infarcts^2^		1.93	0.36–10.47	0.447
CMBs^3^	1.93	1.04–3.59	0.037	CMBs^3^		0.54	0.14–2.03	0.359
Cortical atrophy ^4^	2.45	1.34–4.48	0.004	Cortical atrophy ^4^		0.63	0.24–1.69	0.36
Composite CSVD ^5^	1.5	0.82–2.73	0.186	Composite CSVD ^5^		0.97	0.36–2.63	0.951
Modified STRIVE ^6^	1.56	0.67–3.60	0.301	Modified STRIVE ^6^		1.05	0.33–3.39	0.935

eGFR calculated from creatinine and cystatin C in non-underweight participants and on cystatin C in underweight participants (BMI < 23) using the CKD-EPI formula

eGFR ≥ 60 ml/min/1.73m^2^ as reference

Binary logistic regression models were used to examine the association between CKD (eGFR < 60 ml/min/1.73m^2^) and WMHs, lacunar infarcts, CMBs, cortical atrophy, CSVD composite, and modified STRIVE.

Covariates: Age, sex

*Abbreviation* BMI = Body mass index, CKD = Chronic kidney disease, CKD-EPI = chronic kidney disease epidemiology collaboration, CMBs = Cerebral microbleeds, CSVD = Cerebral small vessel disease, eGFR = estimated glomerular filtration rate, GCA = Global cortical atrophy, MRI = Magnetic resonance imaging, SD = Standard deviation, STRIVE = Standards for reporting vascular changes on neuroimaging, WMHs = White matter hyperintensities

^1^ WMHs defined as Fazekas scale ≥ 2

^2^ Lacunar infarcts defined as presence of ≥ 1 lacunar infarct

^3^ CMBs defined as presence of ≥ 1 CMB

^4^ Cortical atrophy defined as GCA ≥ 1 and/or Koedam score ≥ 1 and/or ≥ 1 frontal/frontotemporal/temporal atrophy

^5^ Presence of CSVD according to the composite CSVD variable was defined as Fazekas scale ≥ 2 and/or presence of ≥ 1 lacunar infarct and/or ≥ 1 CMB

^6^ Presence of CSVD according to the modified STRIVE variable was defined as Fazekas scale ≥ 2 and/or presence of ≥ 1 lacunar infarct and/or presence of ≥ 1 CMB and/or presence of cortical atrophy and/or presence of central atrophy

Results of the stratified analyses are shown in Table 1. No association was observed between CKD and CSVD in the non-hypertensive group. In the hypertensive group, adjusted for age and sex, we observed that CKD was associated with CMBs (OR 1.93, CI 1.04–3.59, p-value 0.037), as well as with cortical atrophy (OR 2.45, CI 1.34–4.48, p-value 0.004).

## Discussion

We have explored the association between CKD and CSVD in a general population cohort of older adults. All markers of CSVD were more prevalent in the CKD group compared to those with normal kidney function. We observed that CKD was associated with CMBs and cortical atrophy in the hypertensive group. No statistically significant associations were observed in the non-hypertensive group.

### Results in previous studies

Previous studies have reported mixed findings regarding an association between CKD and CMBs, though recent pooled meta-analyses support such a connection [17, 18]. Kim et al. [37] found that the level of eGFR impairment was associated to the burden of CMBs, which also is supported by other studies (Shima et al. [38], Van Overbeek et al. [39]). In contrary to our findings, Kim, Shima and van Overbeek all found the link between eGFR and CMBs to be independent of hypertension. In regard to this seemingly inconsistency, a few differences between these studies can be highlighted. Kim et al. used the modification of diet in renal disease (MDRD) formula to estimate eGFR. MDRD is associated with less accuracy compared to the CKD-EPI formula [40], increasing the risk of misclassification of subjects based on kidney function in this study. The participants in the study performed by Shima et al. had more advanced CKD compared to the participants in this study, which represents a relatively healthy older population. The study by Van Overbeek et al. had a longitudinal setting, investigating CKD and progression of CMBs over time.

Not all studies have found an association between CKD and CMBs. Akoudad et al. [41] found no association between eGFR and CMBs. A possible explanation to this could be that lobar CMBs were overrepresented in their material. Lobar CMBs have a stronger association to cerebral amyloid angiopathy than to arteriosclerosis. Neither did Sedaghat et al. [42] find any association between decline in eGFR and incident CMBs over time, with or without adjustments made for hypertension in their regression models. In another recent longitudinal study, Jiménez-Balado et al. [43] also found no association between decline in eGFR and incident CMBs. Even though the estimation of GFR in these two latter studies were measured from creatinine alone, increasing the risk of overestimation of eGFR, the mean eGFR of the participants in both these studies were high, almost 80 ml/min/1.73m^2^, representing relatively healthy populations. It is possible that the follow-up time of 5 and 4 years, respectively, in these two studies was too short to find any associations between decline in eGFR and incident CMBs.

Compared to CKD and CMBs, less is known regarding the connection between CKD and brain atrophy. Similar to our findings, Yakushiji et al. [44] found CKD (eGFR < 60 ml/min/1.73m^2^) to be associated with cerebral atrophy. Chang et al. [45] found mild CKD (defined as eGFR < 90 ml/min/1.73m^2^) to be associated with reduced gray matter volume and cortical thickness in a group of older participants. In contrast to our findings, the connection between CKD and brain atrophy found by both Yakushiji and Chang were independent of hypertension. In another study by Ikram et al. [46], no association between eGFR and cerebral atrophy was found. In all these studies comparing CKD to cerebral atrophy, eGFR was based on creatinine alone, which risks overestimating GFR and making the results more difficult to interpret.

### The role of hypertension

In this study, we observed that CKD was associated with CMBs and cortical atrophy in the hypertensive group. In the non-hypertensive group, no association between CKD and CSVD was observed.

Both the brain and the kidneys are high energy consuming organs, dependent on a high, continuous, and stable blood flow. The vascular resistance in the kidney and the brain are low compared to other organs, resulting in a greater exposure of their vascular beds to pulsatile pressure. This presumably make both the kidney and the brain sensitive to hypertension [13, 14]. Hypertension is strongly related to damage in the microvasculature of the kidneys (hypertensive nephrosclerosis), one of the most common pathways leading to end stage renal disease [25]. Whether CKD is a cause of hypertension or vice versa is not clear and has long been a matter of debate. The matter is not resolved, but there is recent evidence that gives support to the latter claim [47].

Hypertension is also strongly related to CSVD [1], including both CMBs [30] and brain atrophy [48]. A recent longitudinal trial from the Framingham Heart Study, showed that hypertension in mid to late life preceded development of CMBs later in life [49]. Another recent longitudinal study found that diastolic hypertension preceded progression in CVSD [50]. Zhang et al. [51] found an association between reduction in white matter blood flow and WMHs, and hypertension has been associated with reduction in global and regional cerebral blood flow [52]. These arguments indicate that hypertension most likely contributes to the development of CSVD.

Hypertension is heavily associated with arterial stiffness [53]. Arterial stiffness is also associated with CSVD [54, 55], as well as with CKD [56]. Arterial stiffness is associated with elevated pulse wave velocity and elevated pulsatile pressure [53]. The vascular beds of the kidney and the brain are, as mentioned above, proposed as particularly vulnerable to increased pulsatile pressure. Hypertension is also associated with endothelial dysfunction (ED) [57]. ED is related to CKD [58], as well as to CSVD [59]. An association between CKD and markers of CSVD was observed only in the hypertensive group in this study. A hypothesis regarding the pathophysiology behind this association is that hypertension-related damage to the microvasculature of the kidney and the brain is responsible. This hypertension-related damage could depend on elevated pulsatile pressure, or hypertension-related ED, or both.

Hence, an explanation to the associations between CKD and markers of CSVD observed only in the hypertensive group in this study, could be hypertension-related damage to the small vessels in both the kidney and the brain, resulting in impairment in eGFR, as well as CSVD.

### Renal contribution to development of CSVD

Hypertension is related to reduction in function of the blood-brain barrier [60]. A possible implication of renal dysfunction on CSVD in hypertensive subjects could be that a history of hypertension, with a following reduction in the blood-brain barrier function, leaves the brain more susceptible to damage from fluid and electrolyte imbalances linked to CKD [61].

The results in a recent longitudinal mouse study by Lau et al. [62] rises yet another theory. Lau et al. showed that CKD leads to disruption of the blood-brain barrier independently of hypertension. Since CKD was related to CMBs and cortical atrophy only in the hypertensive group in this study, it is possible that the combination of CKD and hypertension, both capable of damaging the blood-brain barrier, resulted in brain damage, but that CKD alone in this relatively healthy population, did not.

Finally, CKD has been associated with arterial stiffness independently of hypertension [56], and to ED independently of blood pressure [63]. Hypothetically, this could imply that CKD is indirectly involved in the development of hypertension and the development of CSVD, through the development of arterial stiffness and/or ED.

### Definition of CKD

GFR has in most previous studies been estimated using the CKD-EPI formula. The most common proteins used to estimate GFR in the CKD-EPI formula are crea and cysC. The use of crea, cysC or a combination of crea/cysC in the formula varies among studies, although the use of crea alone seems to be most common. The use of crea for estimating GFR can be problematic since crea is directly related to muscle mass. In the older population, where loss of muscle mass and sarcopenia is common, crea often overestimates GFR [29]. In a recent large review (*n* = 23 667), Zou et al. found that the use of CKD-EPI based on cysC to estimate GFR was associated with less bias compared to the use of crea or crea/cysC, while the use of crea/cysC provided the highest accuracy [64]. A previous study of the GÅS cohort has found a significant amount of variation between eGFR based on cysC compared to crea using the CKD-EPI-formula (≥ 10% variation was found in 65%, and > 30% variation was found in 19% of the participants) [65]. Low body weight is associated with low muscle mass in older people [66]. In the older population underweight is commonly defined as BMI < 23 [28]. Since using crea/cysC to estimate GFR provided the highest accuracy in the review of Zou et al., eGFR was based on crea/cysC in the non-underweight participants. To minimize bias and overestimation of GFR in underweight participants, eGFR was based on cysC alone in participants with BMI < 23.

In this study, GFR was estimated from cysC alone in underweight participants. There are studies that have found cysC to be a risk factor of CSVD independent of eGFR based on creatinine [17], which theoretically could bias eGFR based on cysC in the underweight of this study. The cause of the association between cysC and CSVD independent of eGFR based on creatinine is not fully understood. CSVD-related release of microvesicles containing cysC has been proposed [67]. Grubb et al. introduced the term shrunken pore syndrome (SPS) in 2015 [68]. In SPS, small molecules such as crea, are assumed to be more easily excreted in the glomeruli of the kidneys compared to larger molecules, such as cysC. SPS is associated with elevated proteins associated with atherosclerosis [69]. To truly determine cysC as a risk factor of CSVD independent of GFR, GFR should not be estimated indirectly through measurement of endogenous substances, but directly by assessing GFR based on excretion of exogenous substances, such as iohexol. To the knowledge of the authors, this has not yet been performed.

A sensitivity analysis was made where all statistical models were re-run with GFR estimated using the CKD-EPI formula based on crea/cysC in all 390 participants (results not shown). When eGFR was based on crea/cysC in all participants, the number of subjects with CKD status decreased from 94 to 87. All estimates and CI:s in the statistical models remained similar. With the statistical significance level set at 5%, all previous associations remained, except for an association that was lost between CKD and CMBs in the hypertensive group adjusted for age and sex (p-value 0.063). It is likely that an association was harder to observe due to the smaller CKD group and dilution of the group with normal kidney function by subjects that, in reality, had CKD.

### Definition of CSVD

WMHs, lacunar infarcts, CMBs, and brain atrophy are commonly regarded as components of CSVD [1, 36]. WMHs, lacunar infarcts, and CMBs are focal lesions, and their definitions do not vary significantly between studies. Brain atrophy can be global or focal, and especially global atrophy represents the outcome of a more diffuse process. The definition of brain atrophy varies extensively, making comparisons between studies more difficult. Brain atrophy can be measured using different visual scales, as the Pasquier scale or the Koedam score. Brain atrophy can also be assessed using automatic brain tissue segmentation (for example of gray matter or of white matter) [70]. In addition, the pathophysiological geneses behind brain atrophy are diverse, and different pathological processes in the central nervous system can lead to brain atrophy. Common pathologies leading to brain atrophy are CSVD and Alzheimer´s disease. Alzheimer´s disease typically leads to atrophy of the medial temporal lobe and/or the parietal lobe. The localizations of brain atrophy as part of CSVD is less studied, but seem to include global cerebral atrophy [71]. Examples of other pathologies that can lead to brain atrophy are excessive alcohol use, Parkinson´s disease, multiple sclerosis, and sleep disorder [70, 72].

### Clinical implications

Since this was a cross-sectional designed exploratory study, no causal relationships could be established. We observed that eGFR was associated with markers of CSVD in the older hypertensive population. Hypertension is an important risk factor of CSVD, and CSVD is heavily linked to major disorders, such as dementia and stroke. The findings of this study emphasize the importance of assessment and management of hypertension in older individuals with CKD.

### Strengths and weaknesses

Strengths of this study include representation of the general, older population, a relatively large sample size compared to other MRI studies, and use of the same experienced neuroradiologist to ensure consistency in the evaluation of the MRI images. Previous studies investigating inter-rater reliability regarding markers of CSVD on MRI, have found a high inter-rater reliability regarding assessment of WMHs, CMBs, and cortical atrophy according to Pasquier scale and the Koedam score [73–76], and a moderate to high inter-rater reliability regarding lacunar infarcts [77].

Another strength of this study includes the use of both crea and cysC to accurately estimate GFR using the reliable CKD-EPI formula, as well as the consideration taken to underweight participants, by estimating GFR only on cysC in this group to minimize bias.

We acknowledge that this study had limitations. An association between CKD and markers of CSVD was observed only in the hypertensive group in this study, and we cannot rule out hypertension as a potential confounder in this relationship. This was, however, an exploratory study. Further studies are warranted to further explore the relationship between CKD, CSVD, and hypertension.

Perivascular spaces and recent small subcortical infarcts are in many studies considered entities of CSVD. Recent small subcortical infarcts are included in the SVD score, and perivascular spaces are included in both the SVD score and in STRIVE [35, 36]. Perivascular spaces and small subcortical infarct were not measured, and therefore a modified version of the SVD score (composite CSVD) and a modified version of STRIVE (modified STRIVE) were created. This could limit the comparability of our findings with other studies.

Compared to those with normal kidney function, the group with CKD were slightly older, and they also had a greater burden of cardiovascular risk factors and events, such as hypertension, diabetes, smoking, coronary artery disease, and heart failure (see Table 2), which most likely contributed to the difference in CSVD burden between the groups.

The logistic regression models were adjusted for age and sex. The number of CSVD related outcomes in the group with CKD was relatively small (see Table 2), and therefore no adjustments for further potential covariates were performed. We acknowledge that the small prevalence of some of the lesions could have prevented us from observing associations between CSVD and CKD because of lack of power. This is especially true regarding lacunar infarcts, where only 10 cases were observed in the CKD group.

The vast majority of subjects in this study had an eGFR ≥ 60 ml/min/1.73m^2^(76%), representing normal kidney function (eGFR mean = 70.9 ml/min/1.73m^2^, eGFR median = 71.6 ml/min/1.73m^2^, eGFR 25 percentile = 60.6 ml/min/1.73m^2^, eGFR 75 percentile = 81.9 ml/min/1.73m^2^). The use of eGFR as a continuous variable could potentially have allowed the exploration of a dose-dependent relationship between eGFR and the severity of CSVD. However, since the vast majority of subjects in this study had normal kidney function, to avoid extrapolation, the variable representing kidney function was kept binary.

Another limitation of this study is that the blood samples were not taken at the MRI examination. To investigate this issue, we performed a sensitivity analysis to identify the CKD status at the next study visit after the MRI visit. The mean time from the MRI examination until the following blood sampling was 605 (SD 399) days. GFR was estimated in the same procedure as above. 212 (52,1%) out of the 407 participants who underwent MRI, continued attending visits in the GÅS study. Out of these 212 participants, 28 (13.2%) had missing GFR status in the following GÅS visit. The vast majority, 147 (69.3%) participants, remained stable in their group based on eGFR. 7 (3.3%) participants went from CKD to normal kidney function, and 30 (14.2%) went from normal kidney function to CKD. We do not know if CKD in these cases appeared before or after the MRI examination. Presumably, some of the participants who had been classified as having normal kidney function, could have had developed CKD before the MRI examination. Therefore, the observed associations between CKD and CSVD in this study could be underestimated.

## Conclusions

We observed an association between CKD and markers of CSVD (CMBs and cortical atrophy) in the general older hypertensive population. In the general older non-hypertensive population, no associations between CKD and any markers of CSVD were observed. Our findings might indicate that hypertension is a link between CKD and CSVD. Further studies investigating the relationship between CKD, CSVD, and hypertension are warranted.

## Data Availability

All data in this study was based on the GÅS dataset. The GÅS dataset is restricted and not publicly available. The data can be obtained by the co-author and GÅS research leader professor Sölve Elmståhl by reasonable request.

## Abbreviations

BMI: Body mass index
CI: Confidence interval
CKD: Chronic kidney disease
CKD-EPI: Chronic kidney disease epidemiology collaboration
CMBs: Cerebral microbleeds
CSVD: Cerebral small vessel disease
DBP: Diastolic blood pressure
DWI: Diffusion-weighted images
ED: Endothelial dysfunction
eGFR: Estimated glomerular filtration rate
IDMS: Isotope-dilution mass spectrometry
GCA: Global cortical atrophy
GFR: Glomerular filtration rate
GÅS: Good aging in Skåne
MRI: Magnetic resonance imaging
SBP: Systolic blood pressure
SD: Standard deviation
SNAC: Swedish national study on aging and care
SPS: Shrunken pore syndrome
STRIVE: Standards for reporting vascular changes on neuroimaging
SVD: Cerebral small vessel disease
SWAN: Susceptibility-weighted angiography
T2 FLAIR: T2-weighted fluid-attenuated inversion recovery
WMHs: White matter hyperintensities
